# Metagenomic next-generation sequencing assists the diagnosis treatment of fungal osteoarticular infections

**DOI:** 10.3389/fcimb.2022.1072539

**Published:** 2022-11-23

**Authors:** Chaofan Zhang, Yunzhi Lin, Changyu Huang, Zida Huang, Xinyu Fang, Guochang Bai, Zeyu Zhang, Wenbo Li, Wenming Zhang

**Affiliations:** ^1^ Department of Orthopaedic Surgery, The First Affiliated Hospital, Fujian Medical University, Fuzhou, China; ^2^ Department of Orthopaedic Surgery, National Regional Medical Center, Binhai Campus of the First Affiliated Hospital, Fujian Medical University, Fuzhou, China; ^3^ Fujian Provincial Institute of Orthopedics, The First Affiliated Hospital, Fujian Medical University, Fuzhou, China; ^4^ Department of Stomatology, The First Affiliated Hospital, Fujian Medical University, Fuzhou, China; ^5^ Department of Stomatology, National Regional Medical Center, Binhai Campus of the First Affiliated Hospital, Fujian Medical University, Fuzhou, China

**Keywords:** fungal osteoarticular infection (FOI), metagenomic next-generation sequencing (mNGS), septic arthritis, periprosthetic joint infection (PJI), osteomyelitis

## Abstract

**Background:**

Fungal osteoarticular infection (FOI) is not commonly seen in clinical practice but proposes a great challenge to orthopedic surgeons. In this study, we aimed to investigate the risk factors, the clinical features, and surgical outcomes of FOI in our institution. Specifically, we aimed to explore the role of metagenomic next-generation sequencing (mNGS) in the diagnosis and treatment of FOI.

**Methods:**

All the patients who were diagnosed and managed with FOI in our institution from January 2007 to December 2020 were retrospectively reviewed, including primary fungal implant-related infection, primary fungal osteomyelitis or arthritis, and fungal infections secondary to bacterial osteomyelitis or implant-related bacterial infections. The potential risk factors and the clinical and surgical features were analyzed. The pathogen data were compared between culture and the mNGS test.

**Results:**

A total of 25 patients were included, namely, 12 primary implant-related infections, 7 primary fungal osteomyelitis or arthritis, and 6 fungal infections secondary to bacterial osteomyelitis or implant-related bacterial infections. Most cases had undergone multiple surgeries or long-term antibiotic treatment. Diagnosis was mainly based on microbial culture and the mNGS test. Optimization of culture methods and the use of mNGS assisted the diagnosis. Specifically, mNGS was performed in 12 patients, 5 of whom were culture-negative. In the remaining seven cases, mNGS demonstrated the same results as culture. Management of FOI was complicated as most patients required multiple surgeries followed by long-term antifungal treatment. In selected cases, antifungal-impregnated cement spacer retention can be an optional choice. The overall success rate was 100% (25/25) for our cohort.

**Conclusion:**

We concluded that patients with comorbidities and a history of multiple surgeries or long-term antibiotics are under higher risk for FOI. Use of mNGS assists the diagnosis and treatment of FOI. Surgery combined with long-term antifungal treatment achieved satisfactory outcomes. In selected cases, antifungal-impregnated cement spacer retention can be an optional treatment choice.

## Introduction

Fungal osteoarticular infections (FOIs) are uncommon but debilitating infections, some of which have become clinically opportunistic pathogens. The diagnosis and management of FOI remain challenging for orthopedic surgeons. Most FOIs had an insidious onset of symptoms and thus were not recognized early (Gamaletsou et al., 2012). Treatment of FOI usually includes multiple aggressive surgeries followed by long-term use of antifungal medication, but the outcome was generally not satisfactory (Miller et al., 2015).

FOI can be primary infection or secondary to bacterial infection. Common routes of infection include direct inoculation, contiguous spread, and hematogenous dissemination (Miller et al., 2015). FOI resulting from fungemia, skin lesion, or trauma was rarely reported (Taxy and Kodros, 2005; Dewar and Sigler, 2010; Lee et al., 2012; Lu et al., 2012; Jeong et al., 2013; Cevik et al., 2016), while FOIs secondary to orthopedic surgeries were more commonly seen in literature, especially in implant-related cases. Implant-related fungal infections, typically fungal periprosthetic joint infection (PJI) after hip/knee arthroplasty, was even more difficult to diagnose and treat for the reason of biofilm formation on the bioprosthetic surfaces (Cobo et al., 2016).

Most studies on FOI were case reports or cohort studies with a limited sample size. Thus, its risk factor, clinical presentation, diagnosis, management, and treatment outcome were not well characterized. In this study, we reviewed all the FOI cases that were diagnosed and treated in our institution. The aim was to investigate the risk factors, the clinical features, and surgical outcomes of FOI in our institution. Specifically, we aimed to explore the role of metagenomic next-generation sequencing (mNGS) in the diagnosis and treatment of FOI.

## Methods

### Study characteristics

This study was approved by the Ethics Committee of our institution in accordance with the Helsinki Declaration of 1975 (as revised in 2008). All the cases that were diagnosed and treated with FOI in our institution were from January 2007 to December 2020. Patient demographic information (age, sex, surgical side, if combined with sinus, past medical history, and surgical history), inflammatory markers, microbial data, and mNGS were traced and recorded.

### Inclusion and exclusion criteria

FOI was defined as previously described by Gamaletsou et al.: (1) compatible clinical characteristics; (2) consistent radiographic features; and (3) isolation of fungus in culture and/or histology from samples of bone tissue or metal hardware obtained by open surgery or percutaneous biopsy (Gamaletsou et al., 2012). Specifically for PJI, diagnostic criteria referred to the Musculoskeletal Infection Society (MSIS) guideline for PJI (Parvizi et al., 2011). All infection types, including primary fungal implant-related infection, primary fungal osteomyelitis or arthritis, and fungal infections secondary to bacterial osteomyelitis or implant-related bacterial infections, were included. Patients with a follow-up time of less than 1 year were excluded from the study.

### Surgical technique

#### Aspiration before surgery

Patients with highly suspected fungal FOI routinely underwent aspiration in a sterile environment before operation under the guidance of ultrasound if necessary. The aspirated synovial fluid or pus was immediately sent for white blood cell (WBC) count, percentage of polymorphonuclear leukocytes (PMNs), and microbial culture, including bacteria (aerobic and anaerobic), fungi, and tuberculosis (TB), as well as an antibiotic susceptibility test and the mNGS test. In case of failed aspiration, a complete set of specimens was collected during the operation and transported immediately by designated personnel for further investigations.

#### Surgical steps

All operations were performed by the same experienced senior surgeon. Generally, the skin and subcutaneous tissue were cut layer by layer to expose the surgical site. The pus, if seen, was aspirated and immediately sent for examinations, including WBC and PMN counts, microbial culture and susceptibility test, and the mNGS test. Thorough debridement would be performed to remove purulent secretions, inflammatory granulation tissue, and scars if they existed. Three to five synovium or granulation tissues with the most obvious inflammatory lesions or inflammatory changes were cut with a scalpel and sent for intraoperative frozen section and pathology examinations, microbial culture, and mNGS if necessary. The surgical site was washed repeatedly. The incision was closed layer by layer. If surgery was performed under arthroscopy, similar debridement procedures were performed. A drain was normally placed and was removed according to the drainage volume.

For patients receiving revision arthroplasty, after debridement and removal of the prosthesis, the surgical instruments, gloves, and gowns were replaced, and re-draping was performed. For single-stage arthroplasty, the new total hip or knee prosthesis was implanted. For two-stage revision, an antibiotic-impregnated cement spacer was inserted. Normally, amphotericin B (200 mg) or vericonazole (300 mg) was added per 40 g of bone cement to prepare the spacer (Chotanaphuti et al., 2019). After surgery, patients were prescribed with systemic antifungal treatment for at least 3 months (Parvizi et al., 2018). During the process, patients were strictly followed up to monitor change of inflammatory markers and any adverse effects of antifungal treatment. The criteria for judging controlled infection were as follows (Parvizi et al., 2013): (1) patient had no infection-related symptoms (incision healed well with no sinus, no active or resting hip or knee pain, no swelling of soft tissue, and normal body and skin temperature); (2) normal inflammatory marker levels [including WBC count, C-reactive protein (CRP) and erythrocyte sedimentation rate (ESR) levels]; and (3) imaging examination did not indicate infection signs, such as bone resorption and osteolysis. After clinical evaluation of the eradication of infection, the second-stage revision surgery was performed. During surgery, the joint fluid and tissue samples were sent for frozen section examination, microbial culture, and the mNGS test if necessary. The spacer was removed, and the new prosthesis was implanted. A drain was placed according to the intraoperative blood loss and was normally removed within 48 h.

### Sample collection and processing

Joint fluid was collected by aspiration preoperatively or intraoperatively as mentioned above. Three to five synovium or granulation tissues with the most obvious inflammatory lesions or inflammatory changes were cut during surgery, avoiding necrotic tissues. Once fluid and tissue samples were collected, they were sent immediately by designated personnel to the Department of Laboratory Medicine for further investigations. For conventional microbiology cultures, samples were stored at room temperature for up to 30 min. The culture duration was usually 5–7 days, but in special cases especially negative culture, it would be extended to 14 days. Samples were stored at −20°C and sent to the molecular laboratory (BGI, Shenzhen, China) for the mNGS test within 24 h. Each sample was divided equally to reduce potential heterogeneity and processed for culture and mNGS in a pairwise way.

### Antifungal drug selection and treatment duration

The selection of antifungal drugs was mainly based on the susceptible test results and was decided after consultation and discussion with infectious disease specialists. If there was no complicated systemic infection, the patient was not given antibiotic treatment before obtaining the fluid or tissue samples. For patients who had been treated with antibiotics in other hospitals, antibiotics were stopped for at least 2 weeks before joint aspiration.

Vancomycin combined with third-generation cephalosporin (ceftazidime) was empirically selected for anti-infection treatment before the results of culture and susceptibility tests were available (Osmon et al., 2013). After obtaining the bacteriology results, which indicates fungal infection, specific antifungal medications were administered. The patients were usually prescribed with intravenous antifungal drugs followed by oral drugs for a total of 6 months, according to the 2013 Consensus Meeting on Periprosthetic Joint Infection (Parvizi et al., 2013) and the 2010 Infectious Diseases Society of America (IDSA) guidelines (JR and WE, 2010). For patients receiving two-stage revision arthroplasty, patients normally received at least 6 months of oral antifungal medication according to the susceptible test results before reimplantation of new prostheses. For polymicrobial infection, as currently there is lack of specific guidelines for antibiotic and antifungal treatment of polymicrobial infections of PJI, choice of antibiotics and antifungal drugs mainly depends on the drug sensitivity test, combined with the consultation opinions of infectious disease specialists and the health status of patients.

### Outcome measurement

The definition of treatment success was that the patient had no local infection-related symptoms at the last follow-up, and with the inflammatory markers returning to normal levels, the imaging examination did not suggest signs of infection (such as prosthesis loosening, bone resorption, or osteolysis), and the surgeons judged that the infection had been eradicated. If the infection relapsed or required one or more operations, or if the patient required lifelong antifungal treatment, the treatment was considered to have failed.

### Statistical analysis

Data on demographic characteristics, clinical and radiological features, microbiology, management, and treatment outcomes were collected and analyzed. Numeric value was presented as mean ± standard deviation (SD). Statistical analysis was performed in GraphPad Prism software (GraphPad Software, Inc., V8.2.0).

## Results

### Demographics

**Figure 1:**
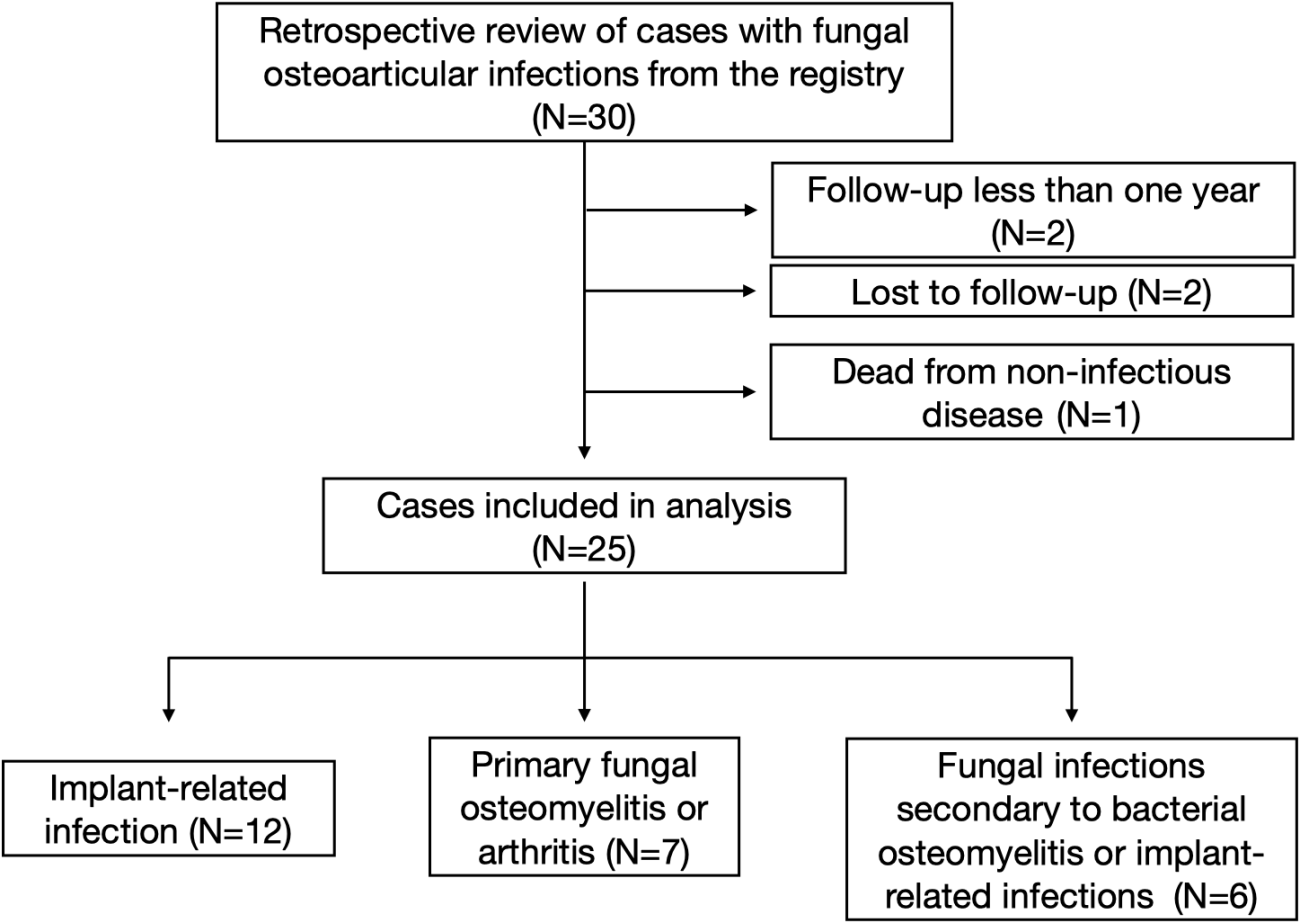
The flow diagram of enrollment of patients.

A total of 30 patients were traced from the registry. Two patients whose follow-up period was less than 1 year was excluded, two patients were lost to follow-up, and one patient died from non-infectious disease, leaving 25 patients included in this study. There were 12 primary implant-related infections [nine after total knee arthroplasty (TKA), one after unicompartmental knee arthroplasty (UKA), one after total hip arthroplasty (THA), one after anterior cruciate ligament (ACL) reconstruction surgery], seven primary fungal osteomyelitis or arthritis (one hip, three knees, one ankle, and two ribs), and six fungal infections secondary to bacterial osteomyelitis or implant-related bacterial infections (two after TKA infections, one after THA infection, two after hip implant infections, and one after femur osteomyelitis). There were 13 female and 12 male patients, with a mean age of 61.7 ± 14.2 (range, 37–88). The mean follow-up time is 40.8 ± 21.7 months (range, 12.0–100.0). A flow diagram was designed and is presented in **Figure 1**. Patients’ demographics is summarized in **Table 1**.

**Table 1:** Demographic information of the 25 patients with fungal osteoarticular infection (FOI).

Case	Type	Sex/age	Underlying condition	Primary diagnosis	Primary surgery	Extra surgery/antibiotics	Fungal species	mNGS	Other pathogens	Sinus	SF WBC (×10^6^/L)	PMN (%)	CRP (mg/L)	ESR (mm/h)	Treatment	FU (months)	Outcome
1	PJI	F/61	HT	TB	TKA (R)	–	*Candida boidinii*	–	–	N	11,889.0	84.3	31.0	64	TR + FCN	25	Success
2	PJI	F/58	HT	OA	TKA (L)	–	*Candida parapsilosis*	–	–	N	10,345.0	60.1	15.8	120	TR + FCN	57	Success
3	PJI	M/62	HT	OA	TKA (L)	–	*Candida albicans*	–	–	N	29,222.0	89.8	<5	72	TR + FCN	57	Success
4	PJI	F/77	DM	OA	TKA (R)	–	*Candida albicans*	–	–	N	17,586	92.9	66	119	TR + FCN	63	Success
5	PJI	F/79	HT	OA	TKA (L)	–	Negative	*Candida tropicalis*	–	N	3,710	70.1	10.9	67	OR + FCN	42	Success
6	PJI	F/37	Hyperthyroidism	TB	TKA (R)	1 DAIR	*Candida parapsilosis*	*Candida*	–	N	3,210.0	82.0	24.2	55	TR + FCN	20	Success
7	PJI	M/67	HT, chronic pneumonia, liver cyst	OA	UKA (L)	–	*Candida albicans*	*Candida albicans*	–	N	1,215.0	62.5	26	79	TR + FCN. Refused reimplantation.	18	Spacer retention
8	PJI	M/45	Renal dysfunction	ONFH	THA (L)	–	*Candida albicans*	–	–	N	52,306.0	71.3	18.6	72	TR + FCN. Refused reimplantation.	66	Spacer retention
9	PJI	F/60	DM, hypoalbuminemia	RA	TKA (R)	Multiple debridement	Negative	*Scedosporium boydii*	–	Y	–	–	<5	40	TR + FCN. Refused reimplantation.	18	Spacer retention
10	PJI	F/81	HT, DM	OA	TKA (L)	–	*Staphylococcus epidermidis* + *Candida parapsilosis*	*Candida parapsilosis*	–	N	–	–	2.77	36	TR + FCN. Refused reimplantation.	48	Spacer retention
11	PJI	F/82	Bilateral pneumonia, tinea pedis	OA	TKA (L)	–	*Candida albicans*	*Candida albicans*	–	N	1,901.0	57	40	70	Long-term voriconazole	12	Success
12	FOI after ACLR	M/37	None	ACL tear	ACLR (R)	–	*Candida albicans*	*Candida albicans*	–	N	110,641.0	80.2	>90	37	Arthroscopic debridement + FCN	24	Success
13	PFOI	M/71	DM, bilateral kidney stone, HBV	Chronic hip arthritis	THA (L)	–	*Candida albicans*	*Candida albicans*	–	N	4,520.0	88.1	108	120	THA + FCN.	31	Success
14	PFOI	M/45	HIV	OA	TKA (R)	–	*Talaromyces marneffei*	–	–	N	299.0	45.8	1.8	2	TKA + FCN.	12	Success
15	PFOI	M/58	None	Septic knee arthritis	None	–	*Fusarium*	–	–	N	35,070.0	79.8	58.4	99	Arthroscopic debridement + voriconazole	54	Success
16	PFOI	M/60	None	Septic knee arthritis	Debridement	Multiple debridement	Negative	*Candida parapsilosis*	–	N	–	–	32.0	78	Arthroscopic debridement + voriconazole	36	Success
17	PFOI	M/65	Pulmonary bullae and emphysema	Ankle TB	None	–	Negative	*Fusarium*	–	N	–	–	18.0	34	Debridement + fusion + voriconazole	18	Success
18	PFOI	F/51	None	Rib malignant neoplasm	None	–	*C. neoformans*	–	–	N	–	–	109	101	Long-term FCN	48	Success
19	PFOI	M/47	DM, HBV	Rib TB	None	–	Negative	*C. neoformans*	–	N	–	–	10.3	41	Long-term FCN	36	Success
20	SFOI	F/80	HT, DM	OA	Bilateral TKA	1 DAIR + debridement + spacer. Long-term abx.	*Cryptococcus laurentii*	–	*Staphylococcus cohnii* subspecies *urealyticum*	N	45,000.0	75.0	10.1	62	Long-term FCN. Refused surgery	100	Spacer retention
21	SFOI	F/88	HT, DM	Intertrochanteric fracture	PFNA (L)	PFNA removal + spacer + debridement. Long-term abx	*Candida albicans*	–	*Staphlycoccus aureus*	Y	–	–	42.0	83	Long-term FCN. Refused surgery	56	Dead
22	SFOI	F/65	Uremia, DM, HT, hypoalbuminemia, anemia	Femoral neck fracture	Cannulated screw fixation (R)	Screw removal + spacer + debridement. Long-term abx	*Candida albicans*	*Candida albicans*	*Enterococcus faecalis*	Y	3780.0	69.1	56	118	Long-term FCN. Refused surgery	55	Spacer retention
23	SFOI	M/63	HBV, DM	OA	TKA (L)	1 DAIR + multiple debridement. Long-term abx.	*Candida tropicalis*	–	*Staphylococcus epidermidis*	Y	2,332.0	33.7	34.0	88	Long-term FCN. Refused surgery	61	Success
24	SFOI	M/52	HT	ONFH	THA (L)	1 debridement + 1 DAIR	*Candida tropicalis*	–	*Mycoplasma hominis*	Y	10,116.0	92.7	22.0	67	Long-term FCN	46	Success
25	SFOI	M/51	None	Osteomyelitis	Debridement	Multiple debridement	*Candida albicans*	–	*Klebsiella pneumoniae*	Y	–	–	10.0	48	Long-term FCN	16	Success

PJI, periprosthetic joint infection; PFOI, primary fungal osteoarticular infection; SFOI, secondary fungal osteoarticular infection; HT, hypertension; DM, diabetes; HIV, human immunodeficiency virus; HBV, hepatitis B; OA, osteoarthritis; TB, tuberculosis; RA, rheumatoid arthritis; ONFH, osteonecrosis of the femoral head; TKA, total knee arthroplasty; UKA, unicompartmental knee arthroplasty; THA, total hip arthroplasty; PFNA, proximal femoral nail antirotation; ACLR, anterior cruciate ligament reconstruction; DAIR, debridement, antibiotics, and implant retention; mNGS, metagenomic next-generation sequencing; abx, antibiotics; SF, synovial fluid; WBC, white blood cell; PMN, polymorphonuclear leukocyte; FU, follow-up; OR, one-stage revision; TR, two-stage revision; FCN, fluconazole.

### History

Twenty-two patients were accompanied with at least one underlying condition, including hypertension, diabetes mellitus, renal dysfunction, hepatitis B, hypoalbuminemia, anemia, and uremia. All the secondary infection cases had undergone multiple surgeries and long-term intravenous antibiotics. Case 7 and Case 14 had previous multiple knee injections. Case 1, Case 6, Case 17, and Case 19 were diagnosed with TB infection and were prescribed with anti-TB treatment for some time. Case 18 was misdiagnosed with malignant neoplasm of the rib (Zhang et al., 2019). Case 11 had tinea pedis, which might be an important source for her fungal knee PJI. Case 14 was a human immunodeficiency virus (HIV) carrier.

### Clinical presentations

Symptoms were insidious in most cases, and progressed slowly. Toxemia symptoms including fever or chills were not commonly seen. In our cohort, only Case 5 presented with fever upon admission. For the primary fungal PJI, patients usually complained of mild pain and swelling of the infected joints. For the primary fungal osteomyelitis or arthritis, patients also complained of mild to moderate pain. In one case (Case 13), the pain even sustained for as long as 2 years. For the infections secondary to bacterial PJI or implant-related infections, patients had undergone multiple surgeries and long-term antibiotics. Patients’ symptoms had subsidized but the tissue or synovial fluid culture showed fungus. Six of the 25 patients had sinus tract, most of which were due to primary bacterial infection.

### Diagnostic procedures

Diagnosis was mainly based on microbial culture of synovial fluid or tissue samples. The mean serum CRP and ESR was 33.5 ± 31.7 mg/L (range, 0–109.0) and 70.9 ± 30.9 mm/h (range, 2.0–120.0), respectively. The mean value of synovial WBC count and polymorphonuclear (PMN) percentage was (20,185 ± 28,383) × 10^6^/L (range, 299.0–110,641.0) and 72.6% ± 16.7% (range, 33.7–92.9), respectively. Galactomannan antigen (GM) test was positive in one case (Case 3). Bone destruction or osteolysis can be seen from the radiographic pictures in some patients (Case 4 and Case 5). CT and MRI scan were also routinely performed to aid the diagnosis.

The microbiology of the 25 patients revealed 10 Candida albicans, 4 Candida parapsilosis, 3 Candida tropicalis, 2 Fusarium, 2 Cryptococcus neoformans, 1 Cryptococcus laurentii, 1 Candida boidinii, 1 Scedosporium boydii, and 1 Talaromyces marneffei. Culture positive rate was 80.0% (20/25). Candida was the dominant fungus species (18/25, 72.0%). For the secondary infections, the original causative organism included one Staphlycoccus aureus, one Staphlycoccus epidermidis, one Staphylococcus cohnii subspecies urealyticum, one Enterococcus faecalis, one Mycoplasma hominis, and one Klebsiella pneumoniae. For Case 8, the culture was negative after 7 days. After extended culture to 12 days, it showed *C. albicans*.

A total of 12 patients received the mNGS test (**Table 1**). Among these patients, six patients demonstrated the same results as culture (Cases 6, 7, 11, 12, 13, and 22). Case 13 was a patient with chronic destructive hip arthritis. The culture and mNGS both demonstrated *C. albicans*, and the patient received one-stage THA. For Case 10, the culture showed multiple specimens with *C. parapsilosis* and one specimen with *Staphylococcus epidermidis*. mNGS demonstrated single reads of *C. parapsilosis*. Considering this, the *S. epidermidis* was deemed as contamination, and the patient received single antifungal treatment, and the follow-up result was excellent. Notably, five cases were with negative culture but mNGS revealed fungal infection. Case 5 was a woman complaining of consistent pain after TKA surgery. Multiple cultures were negative and the patient was initially diagnosed with aseptic loosening. However, the mNGS showed *C. tropicalis*. After consultation with the patient, she received one-stage revision TKA followed by long-term fluconazole treatment. Case 19 was a patient complaining of progressive left back pain for 5 months who was misdiagnosed with TB and received anti-TB treatment. Aspiration under CT scan was performed, and the culture was negative. However, the mNGS showed *C. neoformans* infection. Cases 9, 16, and 17 were also patients in which culture was negative but mNGS showed fungal infection, which assisted the diagnosis and treatment.

### Treatment and outcome

Treatment was difficult as most patients required multiple aggressive surgeries followed by long-term antifungal therapy. The overall success rate was 100%, with no patients who had relapsed infection requiring further surgeries. No patient required lifelong antifungal treatment. One patient had a transient increase in alanine transaminase (ALT) but returned to normal levels without recurrence through short-term drug cessation and liver protection drug treatment. The other patients had no significant adverse effects with long-term antifungal agents.

**Figure 2:**
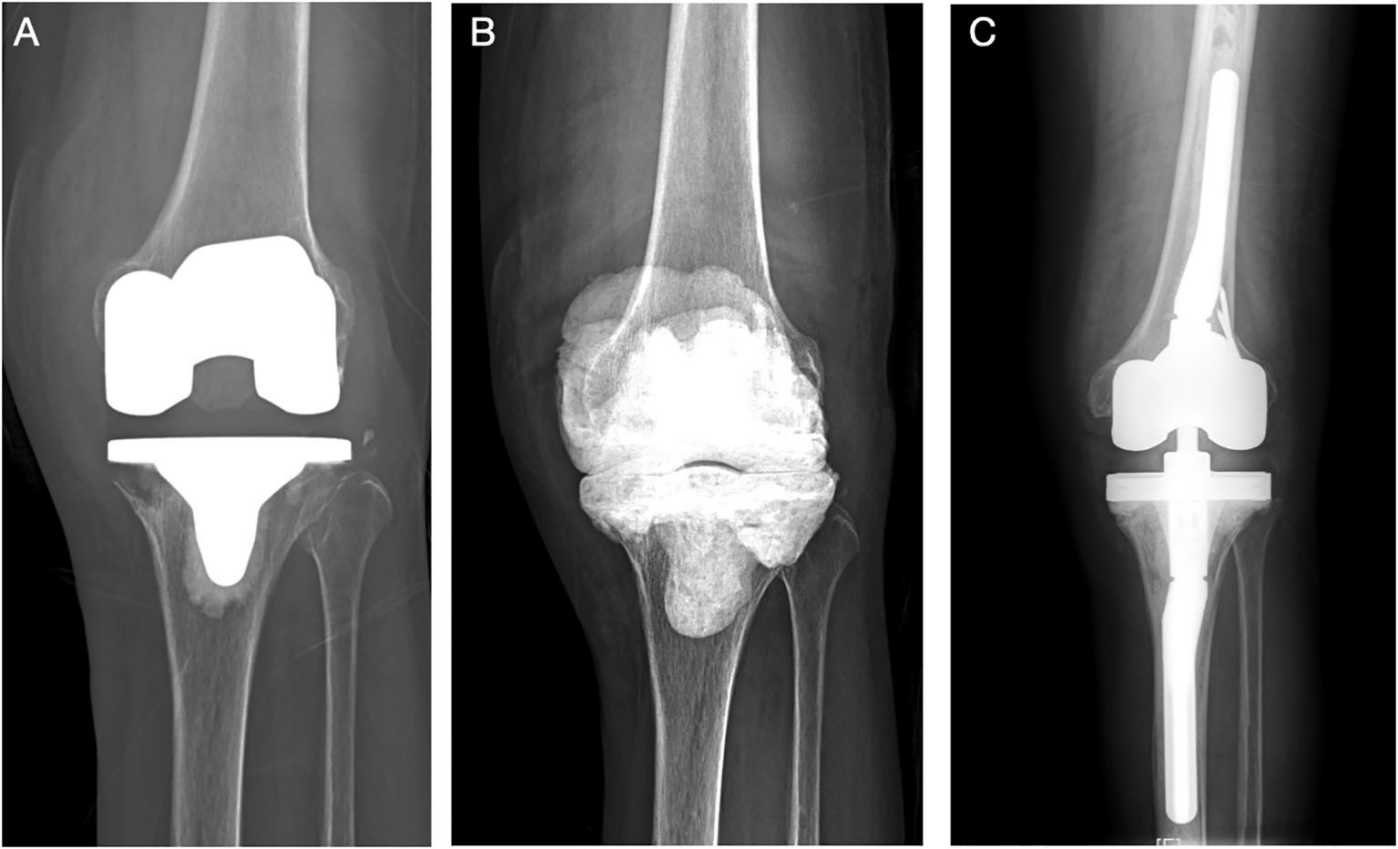
(Case 2) A 58-year-old female patient, with a history of hypertension and diagnosed with knee OA, received left TKA surgery in an outsider hospital. She complained of continuous pain after surgery for 1 year before admitted to our department **(A)**. PJI was highly suspected, and knee aspiration was performed. The synovial WBC count was 10,345.0 × 10^6^/L (PMN 60.1%), and culture showed *Candida parapsilosis*. A two-stage revision surgery was performed **(B)**. The patient received oral fluconazole (300 mg qd) for 6 months, and re-admitted for the second-stage reimplantation at 1 year later. The reimplantation was performed **(C)**. The intraoperative culture was negative. After a 57-month follow-up, no sign of infection was noted.

**Figure 3:**
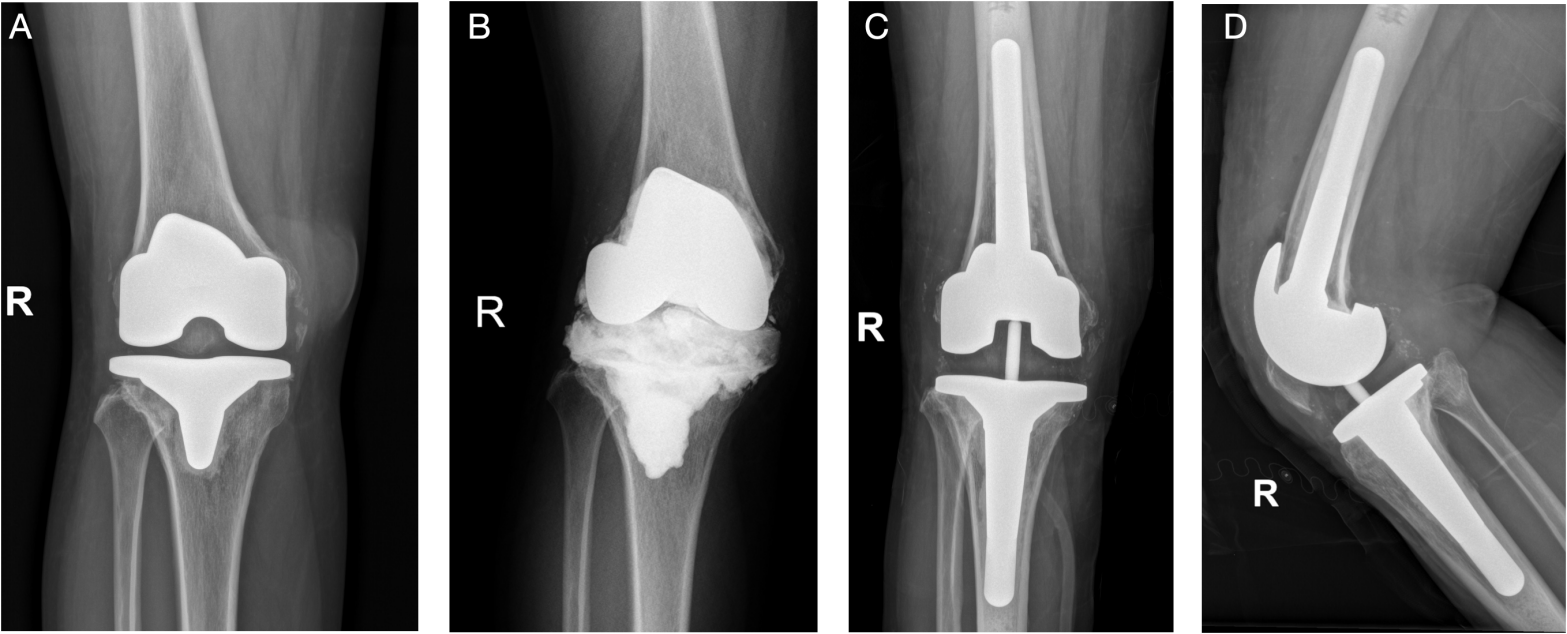
(Case 6) A 37-year-old female patient, with a history of hyperthyroidism, was diagnosed with knee synovitis and received multiple arthroscopic debridement surgeries, followed by TKA surgery, in an outsider hospital. She was later diagnosed with acute PJI and received one DAIR surgery. She complained of continuous pain after surgery and was admitted to our department **(A)**. PJI was highly suspected, and knee aspiration was performed. The synovial WBC count was 3,210.0 × 10^6^/L (PMN 82.0%), and culture showed *Candida parapsilosis*, which was consistent with the mNGS result (*Candida*). A two-stage revision surgery was performed **(B)**. The patient received oral fluconazole (300 mg qd) for 6 months and reimplantation was performed **(C, D)**. After a 20-month follow-up, the position of the prosthesis was satisfactory, and no sign of infection was noted.

**Figure 4:**
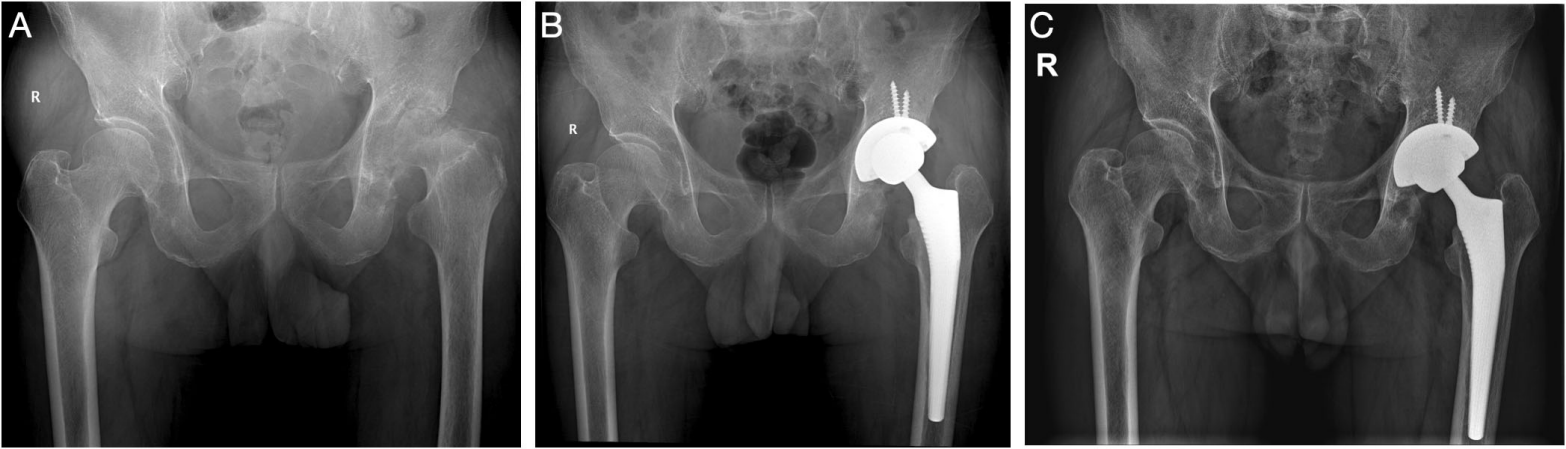
(Case 13) A 71-year-old male patient, with a history of diabetes, bilateral kidney stone, and hepatitis B, complained of left hip pain for 2 years **(A)**. Chronic destructive hip arthritis was highly suspected, and hip aspiration was performed. The synovial WBC count was 4,520.0 × 10^6^/L (PMN 88.1%), and culture and mNGS both showed *Candida albicans*. After consent with the patient, a one-stage THA surgery was performed **(B)**. The patient received oral fluconazole (300 mg qd) for 6 months. After a 31-month follow-up, the patient showed no sign of infection **(C)**.

The 10 primary fungal PJI cases (Cases 1–10) were ordered with revision arthroplasty surgery. All these patients except Case 5, who received single-stage revision TKA, received two-stage revision arthroplasty. Resection arthroplasty was done and an antifungal-impregnated bone cement spacer was implanted. Patients received at least 6 months of oral antifungal medication according to the susceptible test results before reimplantation of new prostheses. Only five patients were re-admitted for the second-stage reimplantation. The culture of synovial fluid and tissues were all negative; thus, mNGS was not performed. For the other four patients, the patients were satisfied with spacer retention and refused to undergo the second-stage reimplantation. The average duration of spacer retention of all patients was 28.4 ± 24.2 months (range, 6.0–72.0). After surgery, patients continued oral susceptible antifungal medication for another 6 months. At the last follow-up, the patients had no sign of relapse. The position of new implant or spacer was satisfactory, with normal inflammatory markers including CRP and ESR. One patient (Case 11) chose conservative treatment and was still under strict follow-up. For the patient with infection after ACL reconstruction (Case 12), after thorough and radical open debridement, followed by long-term antifungal treatment, the patient had no sign of relapse, and the CRP and ESR had returned to normal levels. Three typical cases were presented in **Figures 2**–**4**.

For the seven primary osteomyelitis or arthritis, Case 13 was a patient with chronic destructive hip arthritis. The culture and mNGS both demonstrated *C. albicans*, and the patient received one-stage THA. At the last follow-up, no sign of infection was noted, and the hip function was good, with normal CRP and ESR. Case 14 was a patient with a history of HIV and initially diagnosed with knee OA. However, infection was not completely ruled out before surgery, while puncture failed to aspirate synovial fluid samples. During the TKA surgery, the intraoperative tissue was harvested, which later showed *T. marneffei*. The patient received long-term itraconazole treatment for 1 year. At the last follow-up, no sign of infection was noted, and the knee function was good, with normal CRP and ESR. The patient with primary knee fungal infection of *Fusarium* (Case 15) was managed with thorough and radical arthroscopic debridement surgery, followed by long-term antifungal treatment, and the follow-up results were satisfactory. Case 16 was a patient with septic knee arthritis and received multiple debridement surgery in an outsider hospital. However, all cultures were negative. With the help of mNGS, which showed *C. parapsilosis*, the patient received arthroscopic debridement followed by long-term voriconazole, and was doing great at the last follow-up. Case 17 was presented with ankle pain and swelling for 1 year. Aspiration was performed before surgery; however, the tissue culture was negative. The pathology showed chronic inflammation. The patient was highly suspected of ankle TB and managed with debridement and ankle fusion surgery. However, the mNGS showed *Fusarium* infection. He received long-term antifungal treatment, and the last follow-up showed satisfactory outcome. The two rib infections with *C. neoformans* were prescribed with conservative treatment (Cases 18 and 19) (Zhang et al., 2019). The patients were prescribed with intravenous fluconazole followed by oral fluconazole for a total of 6 months, according to the IDSA guidelines for the management of cryptococcal disease (JR and WE, 2010). At the last follow-up, the patients were asymptomatic and the serum inflammation markers were normal (Zhang et al., 2019).

For the six fungal infections secondary to bacterial PJI, most of them had received multiple surgeries and long-term intravenous antibiotics (**Table 1**). The tissue culture of synovial fluid later showed fungus. These patients received additional long-term antifungal treatment. One patient died from non-infection-related disease (Case 21). At the last follow-up, the patients were asymptomatic and the serum inflammation markers were normal.

## Discussion

Osteoarticular infections due to fungus are rare and represent a diagnostic and therapeutic challenge because no specific guidelines have been established, and published case reports vary widely in therapeutic approach and provide limited information on the microbiological diagnosis. FOI in medical hardware is even more complicated to deal with. To the best of our knowledge, most studies on FOI were case reports or cohort studies with a limited sample size. In the current study, we presented a decent sample size of FOI and report our clinical experience.

### The potential risk factors and clinical characteristics of FOI

Our study showed that multiple underlying diseases, history of multiple surgeries, and long-term use of antibiotics were risk factors for FOI. This is in accordance to the literature. Cobo’s literature review of 73 *Candida* PJI cases showed that 50 patients (68.5%) had systemic disease, 18 (24.6%) had immunosuppressive therapy, 14 (19.1%) had diabetes mellitus, 24 (32.8%) had immunosuppression due to malignant or chronic disease, and 4 (5.4%) had long-term antibiotic use. These patients are believed to be a different type of host with decreased cellular immunity (Azzam et al., 2009). Our study also showed that some patients had multiple joint intrusive operations (such as joint cavity puncture or injection). One patient had tinea pedis of feet, and another case was comorbidized with HIV. These can all be high-risk factors for fungal infection.

FOI had insidious onset of symptoms and can progress slowly. Patients are generally asymptomatic or only mild pain and swelling in the affected joint are involved, as seen in our case series. Cobo’s review of 73 *Candida* PJI showed that only 58.9% and 31.5% of patients complained of pain and swelling, respectively. Fever was only found in 4.1% of patients; 21.9% reported no symptoms (Cobo et al., 2016). Because symptoms are mild and there is frequently no diagnostic suspicion of FOI, the diagnosis can often be delayed. Based on laboratory tests, markers of inflammation in most patients with fungal infections were only moderately to minimally elevated. In our cases, the mean serum CRP and ESR were generally not high (33.5 ± 31.7 mg/L and 70.9 ± 30.9 mm/h, respectively), compared to bacterial infections.

The literature has reported similar findings (Azzam et al., 2009; Gamaletsou et al., 2012; Cobo et al., 2016). The synovial WBC count and PMN percentage varied in our cohorts. The average synovial WBC count and PMN were (20,185 ± 28,383) × 10^6^/L and 72.6% ± 16.7%, respectively, and due to limited cases, we could not draw a conclusion on its value for the diagnosis. Our data were consistent with Azzam’s study (Azzam et al., 2009). These data suggest that in FOI cases, markers of inflammation may not be as high as seen in bacterial infections. Surgeons should take into account the whole clinical picture as well as the local epidemiology of fungus.

### mNGS assists the diagnosis and treatment of FOI

Microbial culture was still considered as the gold standard for diagnosis. However, the main issue is to rule out the possibility of fungal contamination. The dilemma is more pronounced when the culture demonstrates fungal organisms in association with an established bacterial infection (Azzam et al., 2009). Thus, multiple specimens are needed for a wide variety of tests (Hwang et al., 2012). Dark red or blood-stained aspirated fluid may be another hint for fungal infection (Wada et al., 1998). In order to improve the detection rate of fungus, culture duration may have to be extended to 4 weeks after incubation. Several investigators (Brooks and Pupparo, 1998; Selmon et al., 1998; Wada et al., 1998; Ramamohan et al., 2001) have stressed the importance of repeating fluid cultures and obtaining multiple positive tissue cultures before a diagnosis of fungal PJI is made. In one case in our series (Case 8), the culture was negative after 7 days but was positive after extended culture to 12 days. However, even after the optimization of culture methods, the culture positive rate was 80.0% (20/25).

In recent years, with the development of molecular diagnostic technology, mNGS has also been widely used in the diagnosis of infectious diseases (Fang et al., 2020). mNGS directly detects all nucleic acid sequences of the sample by combining high-throughput sequencing with bioinformatic analysis and compares them with the database to identify the species and abundance of pathogens in the samples to be tested. Previous studies have shown that mNGS not only can significantly improve the pathogenic detection rate of bone and joint infection (Huang et al., 2020b; Huang et al., 2020a) but also can be used to guide the special culture method of intraoperative specimens when the routine culture is negative or the bacteriology is not available (Fang et al., 2021). In our series, the mNGS showed consistent results with culture in six patients, and helps rule out one bacterial contamination in one case. Notably, in five patients with negative culture, mNGS all detected fungus. These data provided another evidence that mNGS has potential in assisting the diagnosis and treatment of FOI.

### Surgery combined with long-term antifungal treatment achieved satisfactory outcomes for FOI

Management of FOI conventionally includes multiple aggressive surgeries followed by long-term antifungal therapy. For fungal PJI, surgical options include one-stage revision or two-stage revision surgery. Two-stage revision was a more recognized salvage option, but the success rate varied in literature reports, from 43% to 100% (Wyman et al., 2002; Phelan et al., 2002; Azzam et al., 2009; Dutronc et al., 2010; Anagnostakos et al., 2012; Hwang et al., 2012; Deelstra et al., 2013; Kuiper et al., 2013; Ueng and Lee, 2013; Wang et al., 2015; Geng et al., 2016) (**Table 2**). Cement spacer impregnated with antifungal drugs such as amphotericin B and voriconazole has been used in some institutions and showed promising results (Deelstra et al., 2013; Ueng and Lee, 2013; Wang et al., 2015). However, the efficacy is still under debate (Denes et al., 2012; Miller et al., 2013; Schmidt et al., 2013; Miller et al., 2015), and there have been studies showing that elution of amphotericin B deoxycholate (DAmB) from PMMA is very limited (Kweon et al., 2011). On the other hand, several authors have reported high success rate of one-stage revision for fungal PJI (Selmon et al., 1998; Klatte et al., 2014; Jenny et al., 2016) (**Table 2**). More randomized control studies have to be done to explore which is the better option for managing fungal PJI.

**Table 2:** Success rates of two-stage revision and one-stage revision for fungal PJI.

Reports	Patients	Success rate
Two-stage revision		
Geng et al. (2016)	8	87.5%
Wang et al. (2015)	5	100%
Kuiper et al. (2013)	164	85%
Deelstra et al. (2013)	1	100%
Ueng and Lee (2013)	16	50% (4 dead)
Anagnostakos et al. (2012)	7	100%
Hwang et al. (2012)	30	93.3%
Dutronc et al. (2010)	7	42.9%
Azzam et al. (2009)	19	47.4%
Wyman et al. (2002)	1	100%
Phelan et al. (2002)	4	80%
One-stage revision		
Ji et al. (2017)	11	72.7%
Jenny et al. (2016)	2	100%
Klatte et al. (2014)	10	90%
Selmon et al. (1998)	1	100%

Our cohort also showed satisfactory outcomes of patients receiving either one-stage or two-stage revision arthroplasty. We believe that, to achieve a high success rate in the treatment of FOI, reliable microbial data and standardized surgical techniques are key. First, aspiration before operation should be routinely performed, and the pus should be sent for a set of examinations but not limited to conventional culture. Second, during surgery, thorough and radical debridement of all infected and potentially infected tissues and removal of infected implants and associated foreign material should be carefully performed. Afterward, copious amounts of saline irrigation are required to dilute the bacterial load. Surgical instruments, gloves, and gowns should be replaced, and re-draping should be performed before a new implant is inserted. A second radical debridement was performed thereafter to remove any remnant infective tissues. Meticulous debridement is the basis for success, and we recommend that the procedure be performed by experienced surgeons who have much experience in both single-stage and two-stage revision of PJI.

Another finding of the current study is that spacer retention can also achieve satisfactory outcomes if the patients refused to undergo the second-stage reimplantation. In our series, the average duration of spacer retention of all patients was 28.4 ± 24.2 months (range, 6.0–72.0). The spacer was *in situ* for as long as 6 years in Case 1. Our data were consistent with Choi’s findings, in which they showed 15 out of 18 patients refused the second stage of reimplantation but were all doing well (Choi et al., 2014).

The selection and duration of antifungal drugs following surgery are another question. The benchmark therapies remain amphotericin B and/or fluconazole (Dutronc et al., 2010). Guidelines from the IDSA recommend that *Candida* osteomyelitis be treated with 6–12 months of systemic antifungal agents in conjunction with surgical debridement (Grade-BIII recommendation) (Pappas et al., 2015). The European Society for Clinical Microbiology and Infectious Diseases (ESCMID) guidelines had a similar recommendation (O. A. Cornely et al., 2012). However, resistance of *Candida* species to azole drugs has been reported (Selmon et al., 1998), and there was a new study showing that extended courses of systemic antifungal therapy may be unnecessary when adequate specialized surgical debridement and removal of hardware are performed (Miller et al., 2015). It is important that the selection of the appropriate antifungal treatment is based on antifungal susceptibility testing and a multi-team approach involving infectious disease specialists, clinical pharmacologists, and the treating orthopedic surgeon (Azzam et al., 2009).

Our study found seven cases with primary fungal knee septic arthritis, which is very rarely described in literature (Taxy and Kodros, 2005; Dewar and Sigler, 2010; Gamaletsou et al., 2012; Lee et al., 2012; Lu et al., 2012; Jeong et al., 2013; Miller et al., 2015; Cevik et al., 2016). It usually occurs as a result of accidental implantations of fungus during traumatic procedures, such as surgery, and is usually reported in patients with predisposing factors such as immunosuppression, malignancy, and drug abuse (Lee et al., 2012). Although the majority of knee joint infections are of a pyogenic or tuberculous origin, if a patient complains of mild pain and swelling in the knee and has mild signs of infection, the possibility of fungal infection should be considered (Lee et al., 2012). Primary fungal septic arthritis required joint surgery and lavage to eradicate (Dewar and Sigler, 2010).

### Limitations

There are several limitations of this study that need to be acknowledged, including the limited number of cases, the retrospective nature of the study, and the single-center experience. However, because the incidence of FOI is low, our study, with a decent number of cases, did provide clinical value. This study has described in detail the potential risk factors and the clinical and surgical features of FOI. Our limited experience not only suggested the potential of mNGS as a diagnostic tool for FOI but also suggested that two-stage revision is a useful treatment option for fungal PJI. In selected patients, long-term spacer retention may serve as an alternative option.

## Conclusion

In conclusion, surgeons should always be aware of FOI when treating patients with underlying comorbidities or with a history of multiple surgeries and long-term antibiotics. Diagnosis and treatment of fungal infection remain challenging. Optimization of culture methods and the use of mNGS assist the diagnosis and treatment of FOI. Surgery combined with long-term antifungal treatment achieved satisfactory outcomes. In selected cases, antifungal-impregnated cement spacer retention can be an optional treatment choice.

## Data availability statement

The original contributions presented in the study are included in the article/supplementary material. Further inquiries can be directed to the corresponding author.

## Ethics statement

The studies involving human participants were reviewed and approved by The First Affiliated Hospital of Fujian Medical University. The patients/participants provided their written informed consent to participate in this study. Written informed consent was obtained from the individual(s) for the publication of any potentially identifiable images or data included in this article.

## Author contributions

CZ and WZ conceived and designed the study. CZ, YL, and CH collected the related clinical information, analyzed the data, and wrote the manuscript. ZH, XF, GB, ZZ, WL, and WZ analyzed the data. All authors contributed to the article and approved the submitted version.

## Funding

This work was supported by the Joint Funds for the Innovation of Science and Technology, Fujian province (2019Y9122), the National Natural Science Foundation of China (82102621), the Startup Fund for Scientific Research of Fujian Medical University (2021QH1055), the Talent Introduction Scientific Research Project of the First Affiliated Hospital of Fujian Medical University (YJRC3914), and the Fujian Orthopaedic Bone and Joint Disease and Sports Rehabilitation Clinical Medical Research Center (2020Y2002).

## Conflict of interest

The authors declare that the research was conducted in the absence of any commercial or financial relationships that could be construed as a potential conflict of interest.

## Publisher’s note

All claims expressed in this article are solely those of the authors and do not necessarily represent those of their affiliated organizations, or those of the publisher, the editors and the reviewers. Any product that may be evaluated in this article, or claim that may be made by its manufacturer, is not guaranteed or endorsed by the publisher.
